# Belowground Chemical Interactions: An Insight Into Host-Specific Behavior of *Globodera* spp. Hatched in Root Exudates From Potato and Its Wild Relative, *Solanum sisymbriifolium*

**DOI:** 10.3389/fpls.2021.802622

**Published:** 2022-01-12

**Authors:** Joanna Kud, Syamkumar Sivasankara Pillai, Gabriel Raber, Allan Caplan, Joseph C. Kuhl, Fangming Xiao, Louise-Marie Dandurand

**Affiliations:** ^1^Department of Entomology, Plant Pathology, and Nematology, University of Idaho, Moscow, ID, United States; ^2^Department of Plant Sciences, University of Idaho, Moscow, ID, United States

**Keywords:** *Globodera* spp., root exudates, belowground defenses, *Solanum sisymbriifolium*, potato, pest management, RNA-Seq

## Abstract

Understanding belowground chemical interactions between plant roots and plant-parasitic nematodes is immensely important for sustainable crop production and soilborne pest management. Due to metabolic diversity and ever-changing dynamics of root exudate composition, the impact of only certain molecules, such as nematode hatching factors, repellents, and attractants, has been examined in detail. Root exudates are a rich source of biologically active compounds, which plants use to shape their ecological interactions. However, the impact of these compounds on nematode parasitic behavior is poorly understood. In this study, we specifically address this knowledge gap in two cyst nematodes, *Globodera pallida*, a potato cyst nematode and the newly described species, *Globodera ellingtonae*. *Globodera pallida* is a devastating pest of potato (*Solanum tuberosum*) worldwide, whereas potato is a host for *G. ellingtonae*, but its pathogenicity remains to be determined. We compared the behavior of juveniles (J2s) hatched in response to root exudates from a susceptible potato cv. Desirée, a resistant potato cv. Innovator, and an immune trap crop *Solanum sisymbriifolium* (litchi tomato – a wild potato relative). Root secretions from *S. sisymbriifolium* greatly reduced the infection rate on a susceptible host for both *Globodera* spp. Juvenile motility was also significantly influenced in a host-dependent manner. However, reproduction on a susceptible host from juveniles hatched in *S. sisymbriifolium* root exudates was not affected, nor was the number of encysted eggs from progeny cysts. Transcriptome analysis by using RNA-sequencing (RNA-seq) revealed the molecular basis of root exudate-mediated modulation of nematode behavior. Differentially expressed genes are grouped into two major categories: genes showing characteristics of effectors and genes involved in stress responses and xenobiotic metabolism. To our knowledge, this is the first study that shows genome-wide root exudate-specific transcriptional changes in hatched preparasitic juveniles of plant-parasitic nematodes. This research provides a better understanding of the correlation between exudates from different plants and their impact on nematode behavior prior to the root invasion and supports the hypothesis that root exudates play an important role in plant-nematode interactions.

## Introduction

*Globodera pallida* (Stone, 1972; Behrens, 1975), a potato cyst nematode (PCN) with the potential to cause up to 80% yield loss in potato (*Solanum tuberosum*) (Brodie and Mai, 1989; Contina et al., 2019), is globally one of the most regulated nematode pests (OEPP/EPPO, 2017). Native to South America (Grenier et al., 2010), *G. pallida* has currently spread to other potato-growing regions throughout the world (Dandurand et al., 2019), including the United States (US), where it was first detected in Idaho in 2006 (Hafez et al., 2007). Given the fact that *G. pallida* poses a major threat to the potato industry in the US, which ranks fifth in the global production of this non-grain food crop, strict quarantine measures have been imposed. A new species, *Globodera ellingtonae*, was discovered in Oregon and Idaho in 2008 (Skantar et al., 2011; Handoo et al., 2012). Because its pathogenicity to potatoes remains to be determined, this newly identified nematode is currently not classified as a regulated potato pest in the US (Dandurand et al., 2019). Management of PCN usually combines several integrated approaches such as chemical treatments, crop rotations with a non-host, and early harvesting and trap cropping that allow for the hatching of nematode eggs without the formation of new cysts (Scholte and Vos, 2000). Although the most effective control method against PCN is through the use of resistant cultivars, no commercially accepted potato varieties in the US market carry strong resistance against *G. pallida*. Intensive efforts are being made to identify PCN resistance sources and understand host defenses from wild potato relatives, to transfer them to domesticated potato plants (Castelli et al., 2003, 2006; Whitworth et al., 2018). *Solanum sisymbriifolium* (litchi tomato), a distant relative of tomato and potato, stimulates the hatch of *G. pallida* eggs but does not support nematode reproduction, and therefore can be used as a trap crop (Scholte and Vos, 2000). Little is known about the molecular mechanisms behind *S. sisymbriifolium* immunity, however, a growing body of evidence indicates that both nematicidal properties of biologically active metabolites produced by *S. sisymbriifolium* and a localized cell death possibly mediated through R (resistance) genes may be the major components of the reported PCN immunity (Kooliyottil et al., 2016; Wixom et al., 2020; Pillai and Dandurand, 2021a).

*Globodera pallida* is a highly specialized, obligate, sedentary endoparasite with a complex life cycle. Embryogenesis is followed by the development of the first stage juveniles (J1s) which molt into J2s while still within eggs. The unhatched J2s, protected by a three-layer eggshell are enclosed inside a cyst made of the dead female body, are relatively resistant to chemical and biological stresses. An inner lipid layer of the eggshell is a semipermeable barrier that selectively allows water, small ions, and gasses to pass through (Bohlmann, 2015). Furthermore, the unhatched J2s are partially dehydrated due to osmotic pressure generated by the perivitelline fluid, which has a high content of the disaccharide trehalose in which they are suspended (Perry, 2002). Trehalose-mediated anhydrobiosis induces quiescence and inhibits further molting, but also protects J2s from winter freezing temperatures (Perry, 2002; Duceppe et al., 2017). Because of its narrow host range spanning only plants within Solanaceae, of which potato is the most economically important crop, PCN have developed strategies that limit hatch in the absence of a suitable host, allowing encysted eggs to persist dormant in the soil for decades (Evans and Stone, 1977). To synchronize its lifecycle with the presence of a suitable host, eggs have a nearly absolute requirement for hatching factors, the specific chemical cues from plant root exudates (also called diffusates) released into the soil. The root exudate-stimulated hatching of J2s starts with a calcium-mediated change to the permeability of the inner lipid layer of the eggshell, which is necessary to break dormancy (Clarke and Perry, 1985). An influx of water accompanied by an efflux of trehalose allows J2s to rehydrate and reengage in metabolic processes (Bohlmann, 2015). The hatched J2s exit the cyst and migrate through the soil relying on chemical cues to guide their motion to the root tips of the host (Rasmann et al., 2012). The external sensilla, such as anterior amphids, are assumed to be the main olfacto-sensory organs responsible for the chemoreception of root phytochemicals (Masler and Perry, 2018). These mobile preparasitic J2s do not feed and are solely dependent on their lipid reserves as their source of energy, until they locate a suitable host and induce a feeding site (the syncytium) in the vascular cylinder of roots (Robinson et al., 1987; Bohlmann, 2015). The preparasitic J2s use their stylets to mechanically pierce the cell wall, and release cell wall-degrading enzymes, which are produced in the two subventral gland cells, to chemically facilitate intracellular migration through the outer layers of the root (Davis et al., 2011). Once J2s reach the central cylinder, they select a cambial or procambial cell, which undergoes metabolic transformation resulting in hypertrophy and merges with neighboring plant cells to create the multinucleate syncytium (Bohlmann, 2015). Upon establishment of the permanent feeding site, cyst nematodes become sedentary and complete the remainder of their life cycle inside the root. Proteins, or effectors, secreted by the nematode throughout its parasitic stage enable intracellular migration, formation of a syncytium, and protection from plant defenses.

The plant roots continuously secrete a large number of compounds into the rhizosphere, which modulates the local environment. Root exudates are either transported across the cellular membrane and secreted into the surrounding rhizosphere or produced by root border cells and root border-like cells, which separate from roots as they grow (Hawes et al., 2000; Vicré et al., 2005). The composition of root exudates varies not only depending on the type of plant, but also the type of soil, age, physiological state of the plant, and nutrient availability. The biologically active compounds from root exudates are known to have a multitude of functions in ecological interactions with local microbial soil communities by acting as signaling molecules, attractants, stimulants, and also as inhibitors or repellents. Up until now, only one major hatching factor for *G. pallida*, the triterpenoid solanoeclepin A (SEA), has been identified from the root exudate of potatoes and tomatoes (Schenk et al., 1999). Beyond selective hatching activity, little is known how other root exudate components influence *Globodera* spp. behavior. In this study, we compared the motility, infection, and reproduction rates of *G. pallida* and *G. ellingtonae* J2s hatched in response to root exudate from the susceptible potato cv. Desirée, resistant potato cv. Innovator, and an immune trap crop, *S. sisymbriifolium*, to determine the impact of different solanaceous root exudates on motility, infection, and reproduction on a susceptible potato. To shed light on the molecular mechanisms underlying this differential exudate-mediated modulation of nematode behavior, a transcriptome-wide analysis of hatched J2s was also conducted.

## Materials and Methods

### Root Exudates

Potato plants cv. Desirée and cv. Innovator were clonally propagated in sterile tissue culture conditions. After 14 days, the plants were transferred into 6-inch terracotta pots filled with sterile sandy loam soil and sand (2:1) mix for further growth under greenhouse conditions (18 ± 2°C day-time, 14 ± 2°C night-time, 16:8 h light:dark period). *Solanum sisymbriifolium* seeds (synthetic cross II - SisSynII, obtained from Chuck Brown, USDA-ARS, Prosser, WA, United States) were germinated in peat moss-based germination media and transferred to the sandy loam soil mix under similar greenhouse conditions. The root exudate was harvested by a soil percolation method (Ryan and Devine, 2005) 6 and 8 weeks later. In short, the soil was drenched with deionized (DI) water until saturation and left for 2 h. After that period, additional 200 ml of DI water per pot was added, and the flow-through was collected. The exudate was first filtered through a 0.45 μm filter (Corning, Corning, NY, United States) and then sterilized using a 0.22 μm filter (Corning, Corning, NY, United States). The root exudates were kept at −20°C in the dark for up to 6 months.

### *Globodera* Populations

In this study, two *Globodera* species, *G. pallida* and *G. ellingtonae*, were used. *Globodera pallida* was originally collected in 2006 from an infested potato field in Shelley, ID (Hafez et al., 2007). *Globodera ellingtonae* was obtained in 2008 from an infested field in Powell Butte, OR (Skantar et al., 2011). Nematodes were reared on a susceptible potato cultivar ‘Désirée’ in a greenhouse under standard conditions (10 ± 2°C night-time, 18 ± 2°C day-time,16:8 h light:dark photoperiod). The elutriator method (Byrd et al., 1976) was used to extract cysts from the soil after 16 weeks, dried, and the clean cysts were incubated at 4°C for a minimum of 16 weeks prior to experimental use.

### Hatching of *Globodera* Eggs

The cysts were placed in the meshed polyvinyl chloride (PVC) tubes nested in 6-well plates, surface sterilized with 0.3% hypochlorous bleach for 5 min followed by five rinses in sterile distilled water (Nour et al., 2003), and then hydrated in DI water for 3 days. After 3 days, the water was replaced with root diffusates. The plates were incubated at 19°C and the juveniles (J2s) hatched in the first 10 days of incubation were discarded. The J2s hatched from days 10–14 were collected and used for the study. The Complex Object Parametric Analyzer and Sorter system (COPAS, Union Biometrica Inc., Holliston, MA, United States) was used for sorting uniform J2s (Pillai and Dandurand, 2021b). The sorted J2s were washed with sterile DI water five times and were either used for the infection, reproduction, motility assays, or immediately frozen in liquid nitrogen and stored at −80°C for RNA extraction.

### Plant Inoculation With Juveniles

Two-week-old susceptible potato cv. Desirée plants raised under greenhouse conditions as described above were used for infection and reproduction assays. Each plant was infected with 1,000 J2s hatched in the root exudate from either a susceptible potato cv. Desirée, a resistant potato cv. Innovator, or an immune plant *S. sisymbriifolium*, by directly pipetting the J2s into the soil close to the root zone, approximately 1–1½ inches deep from the surface of the soil. Six plants each were used for the infection and reproduction assays and the experiments were repeated three times.

### Infection and Reproduction Assay

To evaluate the infection rate, the plants were carefully removed from the soil 14 days postinoculation with J2s. The roots were washed over a 200 μm sieve, bleached, and stained with acid fuchsin (Byrd et al., 1983). The stained roots were evaluated under a KL300 LED stereo microscope (Leica Microsystems, Wetzlar, Germany) and all developmental stages were counted to assess infectivity. To assess the reproduction, six infected plants for each root exudate group were grown under greenhouse conditions for a total period of 8 weeks post J2 inoculation. After that, the soil was left to dry completely, and the elutriator method (Byrd et al., 1976) was used to extract cysts from the dried soil. The experiment was repeated three times.

### Motility Assay

The motility assay was conducted in 50 mm sterile Petri dishes filled with 1% Phytagel (Sigma Aldrich, St. Louis, MO, United States). Each plate was divided into three circular zones (zone 1 = 1 cm diameter, zone 2 = 3 cm diameter, zone 3 = 5 cm diameter; see Figure 4 for details). Around one hundred hatched J2s, in 5 μl total volume of H_2_O, were applied in the center of the plate (center of all three circular zones) and their distribution in different zones was evaluated after 90 min under a KL300 LED stereo microscope (Leica Microsystems, Wetzlar, Germany). The experiment was repeated three times.

**FIGURE 1:**
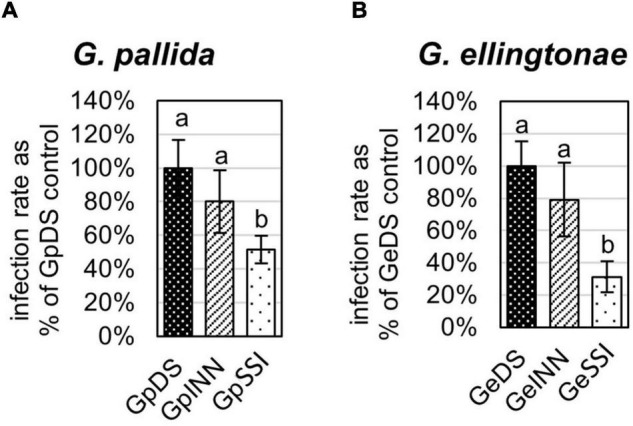
Infection rate of **(A)** *Globodera pallida* or **(B)** *Globodera ellingtonae* on a susceptible potato cv. Desirée (DS) was measured as the percentage of value for a control DS hatching treatment. The number of infecting nematodes of both species was determined 14 days postinfection with J2s hatched in root exudates from susceptible potato control (cv. Desirée - DS), resistant potato (cv. Innovator - INN), or immune plant (*Solanum sisymbriifolium* - SSI). Each value represents the mean ± SE from bulking samples of three independent experiments. Means having the same letter designation are not significantly different as determined using ANOVA followed by Tukey’s HSD test at *p* < 0.05.

**FIGURE 2:**
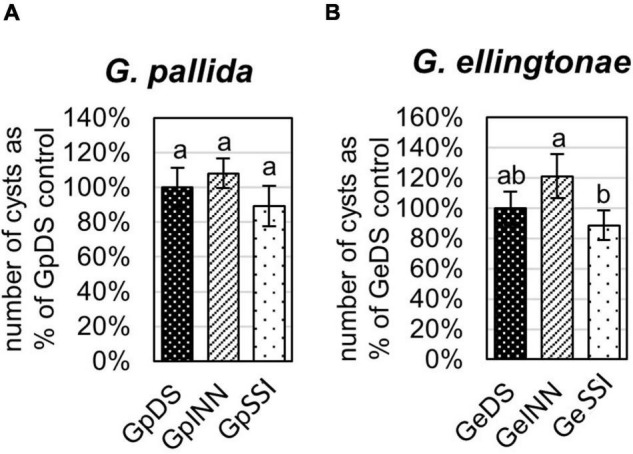
Reproduction rate of **(A)** *Globodera pallida* or **(B)** *Globodera ellingtonae* on a susceptible potato cv. Desirée (DS) was measured as the percentage of value for a control DS hatching treatment. The number of cysts/plant was counted 8 weeks postinfection with J2s hatched in root exudates from susceptible potato control (cv. Desirée - DS), resistant potato (cv. Innovator - INN), or immune plant (*S. sisymbriifolium* - SSI). Each value represents the mean ± SE from bulking samples of three independent experiments. Means having the same letter designation are not significantly different as determined using ANOVA followed by Tukey’s HSD test at *p* < 0.05.

**FIGURE 3:**
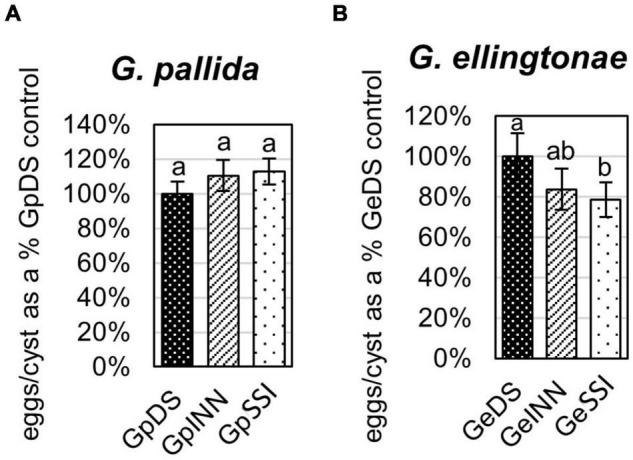
The number of eggs/cyst for **(A)** *Globodera pallida* or **(B)** *Globodera ellingtonae* was measured as a percentage of value for a control DS hatching treatment (susceptible potato cv. Desirée) compared with resistant potato (cv. Innovator - INN), or immune plant (*S. sisymbriifolium* - SSI). Each value represents the mean ± SE from bulking samples of three independent experiments. Means having the same letter designation are not significantly different as determined using ANOVA followed by Tukey’s HSD test at *p* < 0.05.

**FIGURE 4:**
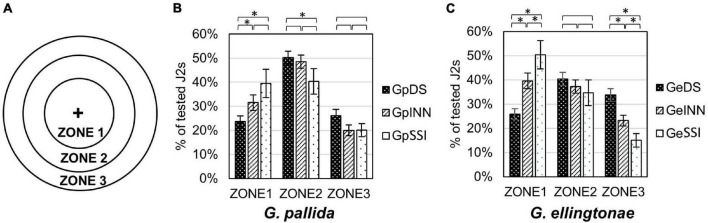
Motility assessment for **(B)** *Globodera pallida* or **(C)** *Globodera ellingtonae* J2s hatched in root exudates from susceptible potato control (cv. Desirée - DS), resistant potato (cv. Innovator - INN), or immune plant (*S. sisymbriifolium* - SSI). **(A)** Graph shows designated zones 1, 2, and 3 in a Petri dish (diameter 1, 3, and 5 cm, respectively). **(B,C)** Numbers represent the percentage of J2s in each zone recorded 90 min after dispensing the desired number of J2s in the middle of a Petri dish plate (“+”). Each value represents the mean ± SE from bulking samples of three independent experiments. Asterisk mark represents significantly different results as determined using ANOVA followed by Tukey’s HSD test at *p* < 0.05.

### RNA Extraction and Sequencing

Each sample of 10,000 J2s was suspended in 650 μl of modified lysis buffer RLT Plus (Qiagen, Hildren, Germany) (1% v/v β-Mercaptoethanol (Sigma Aldrich, St. Louis, MO, United States), 0.2% v/v Antifoam A concentrate (Sigma Aldrich, St. Louis, MO, United States), 20 U/ml SUPERaseIN (Invitrogen, Carlsbad, CA, United States), 20 μg/ml Proteinase K (Invitrogen, Carlsbad, CA, United States). The nematodes were disrupted with one 6 mm Grinding Satellites (OPS Diagnostics, Lebanon, NJ, United States) and 200 μL of 1 mm zirconium beads (OPS Diagnostics, Lebanon, NJ, United States) in 2 ml tubes using a Biospec 3110Bx Mini-Beadbeater-177 1 (BioSpec Products, Bartlesville, OK, United States) at 4,800 oscillations/min for three 20 s intervals. Total RNA was extracted using RNeasy Mini Kit Plus (Qiagen, Hildren, Germany) according to the manufacturer’s instruction and stored at −80°C. RNA integrity assessment and sequencing were carried out at the GENEWIZ sequencing facility (South Plainfield, NJ, United States). The RNA samples with a RNA integrity number (RIN) value of ≥7 were used for the library preparation. The libraries were generated using TruSeq Stranded mRNA Library Prep Kit (Illumina, San Diego, CA, United States) and a paired-end (2 × 150 bp) sequencing was done using the HiSeq2500 (Illumina, San Diego, CA, United States).

### Sequences Processing

Raw reads were processed using TRIMMOMATIC 0.39 (Bolger et al., 2014) to remove low-quality base calls from the reads. Trimmed reads were mapped to the *G. pallida* and *G. rostochiensis* genomes (assembly version Gp_Newton_v1.0 and nGr.v1.1, respectively) (den Akker et al., 2016; Varypatakis et al., 2020) using STAR 2.7 with default parameters (Dobin et al., 2013).

### Quantitative and Functional Analysis

Counting of the mapped reads was performed using HTSeq-count 0.11.2 software with default parameters (Anders et al., 2015). Statistical analysis, including normalization and differentially expressed gene (DEG) identification, was made using the DESEQ2 Bioconductor package in R (Love et al., 2014). The DEGs were identified using a parametric Wald test (*p*adj < 0.01) and a log_2_ fold change (log_2_FC) ≥ 1.5. The Blast2GO bioinformatics platform was used to perform the gene ontology analysis (Götz et al., 2008).

### cDNA Synthesis and qRT-PCR

Total RNA, previously isolated for RNA-seq, was used for the first-strand cDNA synthesis. 250 ng of total RNA was converted into cDNA with SuperScript III reverse transcriptase (Invitrogen, Carlsbad, CA, United States) using Anchored Oligo(dT)_20_ (Invitrogen, Carlsbad, CA, United States) as primers. The Real-Time Quantitative Reverse Transcription PCR (qRT-PCR) was carried out on the Applied Biosystems ViiA 7 system using Power SYBER Green Mater Mix (Applied Biosystems, Foster City, CA, United States). The results were normalized using the *G. pallida EF1*α gene as an internal control (Thorpe, 2012), and the relative expression of target genes (GPALN_014919, GPALN_006587, and GPALN_003867) was calculated based on the comparative 2^–ΔΔCT^ method (Livak and Schmittgen, 2001) using QuantStudio™ Real-Time PCR Software v1.3 (Thermo Fisher Scientific, Waltham, MA, United States). For sequence-specific qRT-PCR analysis, primers were designed using PrimerQuest™ Tool (Integrated DNA Technologies, Coralville, IA, United States) (refer Supplementary Table 2).

### Statistical Analysis

For infection, reproduction, egg viability, and motility assays, a one-way parametric ANOVA in combination with Tukey’s honestly significant difference (HSD) test was performed (*p* < 0.05). For the transcriptome analysis, a parametric Wald test (*p*adj < 0.01) was used to identify significant differences for DEGs. For qRT – PCR analysis, a one-way parametric ANOVA in combination with Tukey’s HSD test was used (*p* < 0.05).

## Results

### Effect of Root Exudate Type on *Globodera* spp. Infection and Reproduction on Susceptible Potato

The infection rate on a susceptible host of J2s hatched in response to root exudate from *S. sisymbriifolium* (SSI) was significantly lower compared with juveniles hatched in potato root exudates (cv. Desirée – DS, or cv. Innovator – INN) regardless of the *Globodera* spp. The decrease in the number of all developmental stages found within the potato roots was greater for *G. ellingtonae*, with a 67% reduction of the infection rate compared to the positive control DS (Figure 1B), while the infection rate of *G. pallida* was reduced by 47% (Figure 1A). Although a slightly lower infection rate was also observed for juveniles hatched in the root exudates of resistant potato (INN) compared with a susceptible DS control, the difference was not statistically significant. The impact of different hatching conditions on reproduction rate, measured as the number of progeny cysts formed, was not significantly different in *G. pallida*, but was significantly different for *G. ellingtonae* hatched in SSI root exudate compared with INN root exudate (Figure 2B). Furthermore, *G. ellingtonae* cysts formed by juveniles hatched in SSI root exudate had slightly fewer eggs than cysts from DS control treatment (Figure 3B). The impact of root exudates on the reproduction rate and the number of eggs per cyst for *G. pallida* was negligible (Figures 2A, 3A). No significant difference in the viability of eggs from progeny cysts was noticed for either *Globodera* spp. (Supplementary Table 1).

### Effect of Root Exudate on the Motility of Juveniles

The root exudates had an impact on the motility of hatched juveniles of both *Globodera* spp. when measured as their ability to disperse in Phytagel over time (Figure 4A). The nematodes that were hatched in either Innovator or *S. sisymbriifolium* root exudates resulted in a less scattered distribution of juveniles compared with the DS control. For *G. ellingtonae*, J2s tended to aggregate close to the loading region with a distribution of 40% in Zone 1, 37% in Zone 2, and 23% in Zone 3 for INN-hatched J2s and 50% in Zone 1, 35% in Zone 2, and 15% in Zone 3 for SSI-hatched J2s, while 26% of DS-hatched J2s was detected in Zone 1, 40% in Zone 2, and 34% in Zone 3 (Figure 4C). *Globodera pallida* juveniles also showed a similar patterns for each zone, with fewer DS-hatched J2s staying in the center Zone 1 (24%) compared with INN (32% of tested J2s) and SSI (40% of tested J2s) (Figure 4B).

### Transcriptomic Insight Into the Root Exudate-Mediated Impact on Hatched Juveniles

**FIGURE 5:**
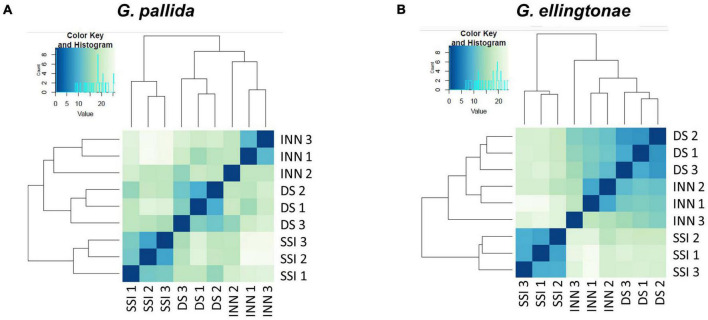
Heatmaps showing the similarity of the different transcriptome libraries for **(A)** *Globodera pallida* and **(B)** *Globodera ellingtonae*. Euclidean distance between samples is based on normalized read counts from DESEQ2 clustered using the heatmap function in R. The darker blue color indicates a closer correlation of expression levels between RNA-seq libraries. Three biological replicates were used for each hatching condition: DS – susceptible potato variety cv. Desirée, SSI – immune plant *S. sisymbriifolium*, and INN – resistant potato variety cv. Innovator.

**FIGURE 6:**
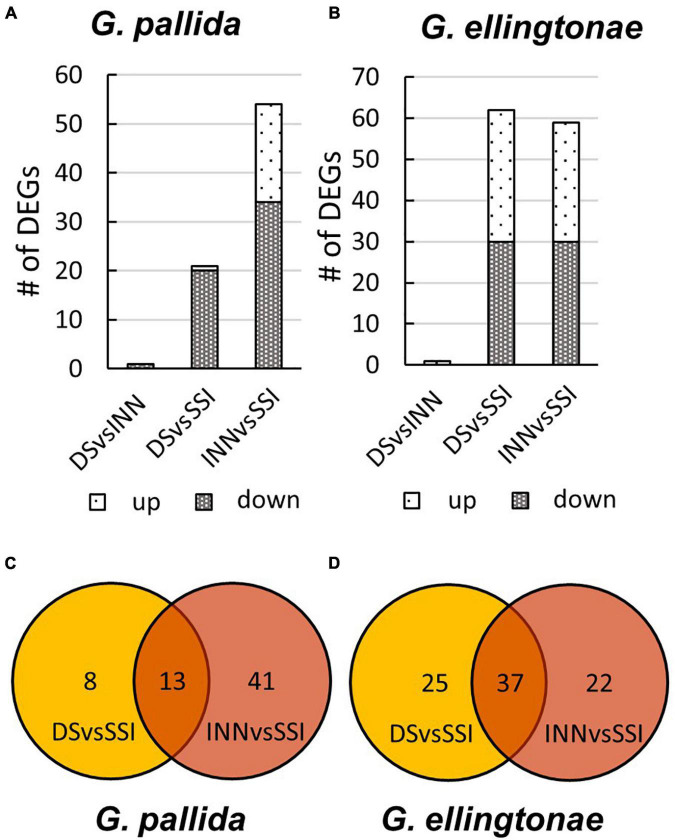
The number of differentially expressed genes (DEGs) for **(A)** *Globodera pallida* or **(B)** *Globodera ellingtonae* based on a comparison between different hatching conditions: DS - susceptible potato control cv. Desirée, INN - resistant potato cv. Innovator, and SSI - an immune plant *S. sisymbriifolium*. The overlap of DEGs identified between DS vs SSI and INN vs SSI comparisons for **(C)** *G. pallida* and **(D)** *G. ellingtonae*.

**FIGURE 7:**
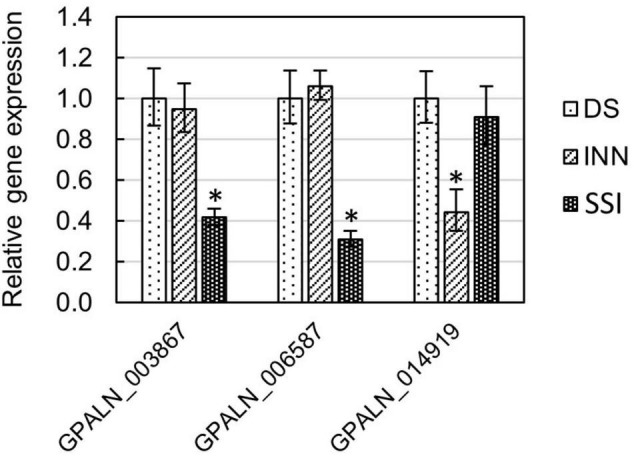
Relative gene expression of selected *Globodera pallida* DEGs (GPALN_003867, GPALN_006587, and GPALN_014919) was measured using the qRT-PCR method. Three different hatching conditions were tested: DS – susceptible potato variety cv. Desirée, SSI – immune plant *S. sisymbriifolium*, and INN – resistant potato variety cv. Innovator. The *Gp*EF1α gene (GPALN_011311) was used as an internal reference gene. Error bars are the mean ± SE, *n* = 3 biological replicates. Asterisk mark represents significantly different results as determined using ANOVA followed by Tukey’s HSD test at *p* < 0.05. See Supplementary Table 2 for primers used.

To ascertain the molecular basis for the observed changes to the nematode behavior, we carried out an RNA-seq analysis of *Globodera* spp. hatched by the three types of root exudates. The RNA- seq yielded an average of 93.76 M paired-end reads (2 × 150 bp) per sample, spanning from 88.48 to 132.58 M (Table 1). The percentage of the *G. pallida* reads that were successfully mapped to the *G. pallida* genome was on an average 90%, whereas the alignment rate for *G. ellingtonae* reads was only 67% (data not shown). Since the published genome of *G. ellingtonae* (Phillips et al., 2017a) has not been annotated, we based our downstream analysis on mapping to the *G. rostochiensis* genome (den Akker et al., 2016). Consistent with previous reports showing more similarities between *G. ellingtonae* and *G. rostochiensis* rather than *G. pallida* (Handoo et al., 2012; Zasada et al., 2013; Phillips et al., 2017b), significantly higher numbers of *G. ellingtonae* reads (average of 80%) were mapped using *G. rostochiensis* genome (Table 1). Heatmaps showed consistent grouping for all the three tested replicates for each root exudate treatment (DS, INN, and SSI) for both *Globodera* spp. (Figure 5). Based on a log_2_ fold change (log_2_ FC) ≥ 1.5 at an adjusted significance value of *p* ≤ 0.01, DEGs were identified by comparing hatching conditions as follows: i. susceptible potato (DS) *vs.* resistant (INN) potato, ii. susceptible potato (DS) *vs.* immune plant (SSI), and iii. resistant potato (INN) *vs.* immune plant (SSI). Whereas a comparison between susceptible and resistant potato root exudates (DS *vs.* INN) yielded only one DEG for each *Globodera* spp., the expression of 21 and 54 *G. pallida* genes was significantly different when analyzing DS *vs.* SSI and INN *vs.* SSI hatching conditions, respectively (Figures 6A,B and Supplementary Data Sheet 1). Similarly, *G. ellingtonae* differentially expressed a larger number of genes when an immune plant was included in the comparison, with 62 DEGs for DS *vs.* SSI, and 59 DEGs for INN *vs.* SSI (Figure 6B and Supplementary Data Sheet 2). However, only 13 genes for *G. pallida* and 36 genes for *G. ellingtonae* met our DEG criteria and overlapped in both comparisons: DS *vs*. SSI and INN *vs*. SSI (Figures 6C,D). Among the DEGs in SSI root exudate-induced hatching, 16 *G. ellingtonae* and 13 *G. pallida* genes showed resemblance to known nematode parasitism genes. All 13 *G. pallida* DEGs were downregulated in the immune root exudate raised J2s while 13 genes were down regulated and three genes were upregulated in *G. ellingtonae* (Table 2). Genes with predicted function in the oxidative stress responses and xenobiotic metabolism represent another group of SSI-specific DEGs (Table 3). A subset of three DEGs (GPALN_014919, GPALN_006587, and GPALN_003867) has been selected for qRT-PCR validation of the RNA-seq data. The expression of tested genes showed a similar profile to the expression pattern obtained through RNA-seq analysis (Figure 7).

**TABLE 1:** Sequencing yield, trimming, and mapping statistics.

Sample ID*	Sequenced reads (M)	Reads after trimming (M)	Mapped (%)**
GpDS1	93.18	83.83	91.05%
GpDS2	92.42	83.10	91.10%
GpDS3	96.04	87.30	86.68%
GpSSI1	92.59	86.01	90.56%
GpSSI2	90.81	81.24	90.05%
GpSSI3	98.10	87.89	89.10%
GpINN1	88.99	79.82	90.94%
GpINN2	112.65	100.67	90.30%
GpINN3	132.58	119.10	89.61%
GeDS1	92.76	84.01	80.46%
GeDS2	94.51	85.36	80.60%
GeDS3	95.35	85.80	80.14%
GeSSI1	88.48	79.54	80.57%
GeSSI2	94.10	85.10	80.45%
GeSSI3	93.45	84.27	80.33%
GeINN1	94.89	85.51	80.13%
GeINN2	96.64	87.58	80.30%
GeINN3	93.65	84.51	80.71%

*Gp, Globodera pallida; Ge, Globodera ellingtonae; DS, susceptible potato variety cv. Desirée; SSI, immune plant S. sisymbriifolium; INN, resistant potato variety cv. innovator.

**% of trimmed reads mapped to G. pallida genome (G. pallida reads) and G. rostochiensis genome (G. ellingtonae reads).

**TABLE 2:** Predicted parasitism genes differentially regulated in SSI hatching treatment compared with potato hatching treatments (resistant - INN or susceptible - DS).

Gene type	*Globodera ellingtonae* DEGs	*Globodera pallida* DEGs	Predicted function
**Downregulated**			
Galectin	GROS_g00816 GROS_g00815 GROS_g02466 GROS_g01645		Camouflage and/or interference with host DAMP signaling (Price, 2019)
C-lectin	GROS_g11229	GPALN_001299 GPALN_001135 GPALN_001300 GPALN_003867 GPALN_001298	
Cellulase	GROS_g07446 GROS_g10505 GROS_g11008	GPALN_015248 GPALN_010598	Cell wall degradation (den Akker et al., 2016)
Expansin	GROS_g03476 GROS_g10585	GPALN_010165	
Transthyretin-like protein	GROS_g07852		ROS scavenging (Lin et al., 2016)
Serine carboxypeptidase	GROS_g02373		Unknown (Huang et al., 2017)
Similar to putative gland protein G20E03		GPALN_015214	Unknown (Klink et al., 2009; Thorpe et al., 2014)
Small effector family in *Globodera pallida*	GROS_g12709	GPALN_005097 GPALN_005105 GPALN_005081 GPALN_005082	Unknown (Thorpe et al., 2014)
**Upregulated**			
Similar to GLAND15	GROS_g02471 GROS_g02470		Unknown (Noon et al., 2015)
Invertase	GROS_g05724		Nutrient metabolism (Danchin et al., 2018)

**TABLE 3:** Differentially regulated genes in SSI hatching treatment compared with potato hatching treatments (resistant - INN or susceptible - DS), predicted to have function in detoxification processes.

Gene type	*Globodera ellingtonae* DEGs	*Globodera pallida* DEGs	Predicted function
Molecular chaperone	GROS_g08159	GPALN_010189	Protein folding (Pérez-Morales and Espinoza, 2015)
Glutathione synthetase	GROS_g09037 GROS_g09038		ROS scavenging (Forman et al., 2009)
THI4 thiazole biosynthetic enzyme		GPALN_010902	ROS scavenging (Duceppe et al., 2017)
Oxidoreductase		GPALN_001038	Xenobiotics metabolism (Hartman et al., 2021)
UDP-glucuronosyltransferase		GPALN_002696 GPALN_003745	
ABC transporter		GPALN_005765	
unknown		GPALN_002494	Unknown, higher expression in response to nematicide Dazomet (Lennon, 2018)

## Discussion

Although root exudates and secretions are a heavy burden on plant metabolism, accounting for a release of up to 40% of photosynthetically fixed carbon (Badri and Vivanco, 2009), they are an important survival mechanism that actively shapes the interaction between plants and their soilborne symbionts and pathogens (Pascale et al., 2020). The detailed chemical composition of root exudates remains hard to determine due to the diversity and complexity of plant secondary metabolites. In the past two decades, a major effort was directed for identifying the unique *G. pallida* hatching factors and study chemicals involved in nematode orientation toward its host plant (Twomey, 1995; Perry and Gaur, 1996; Schenk et al., 1999; Devine and Jones, 2000; Devine et al., 2001; Ryan and Devine, 2005; Farnier et al., 2012; Fleming et al., 2017; Sikder and Vestergård, 2020). The current study is an effort to increase our understanding of the impact of root exudates on nematode behavior prior to the root invasion. Specifically, we compared three hatching conditions including root exudates from i. a susceptible potato, ii. a resistant potato, and iii. an immune plant (trap crop). Past transcriptional studies on *G. pallida* uncovered early changes to gene expression associated with breaking cyst dormancy and hatching in response to tomato and potato root exudate (Duceppe et al., 2017) as well as the comparison of a transcriptional blueprint of hatched *G. pallida* J2s with juveniles of other PCN species *G. rostochiensis* and two non-PCN species, *Globodera tabacum* and *Globodera mexicana* (Sabeh et al., 2019). Additionally, the *G. pallida* transcriptome was analyzed during an early compatible and incompatible interaction with different hosts (Kooliyottil et al., 2019). To our knowledge, this is the first study that shows genome-wide root exudate-specific transcriptional changes in hatched preparasitic juveniles of plant-parasitic nematodes.

Consistent with the previous reports that nematode resistance in potatoes is not attributed to its ability to reduce *Globodera* hatch (Turner and Stone, 1981), the exposure to root exudate from either susceptible or resistant potato induced hatching of a similar proportion in both tested *Globodera* spp. (data not shown). Even though the PCN hatching factor SEA was not reported in *S. sisymbriifolium* root exudate (Sasaki-Crawley, 2012), root exudates from this plant are nearly as effective as potato root exudate for inducing PCN hatch (Scholte, 2000; Ngala et al., 2021), implying the presence of different hatching factors from those produced by potato roots, or concentrations below detection levels. Reflecting only minor changes to nematode behavior between J2s hatched in root exudate from a resistant potato and a susceptible cultivar, our RNA-seq analysis identified only one DEG for each *Globodera* spp. when comparing these hatching conditions (GPALN_014919 for *G. pallida* and GROS_g04684 for *G. ellingtonae*). Both of these genes have a signal peptide and they lack a transmembrane domain suggesting their potential role as secretory proteins (effectors), however, further research is needed to uncover their involvement in parasitism.

The infection rate, however, was greatly reduced when *Globodera* spp. hatch was stimulated by root exudates of the immune plant, *S. sisymbriifolium*, which was supported by our RNA-seq analysis yielding a larger number of DEGs. Recent research on plant-parasitic nematode, *Pratylenchus coffeae*, showed that the expression of two genes for cell wall-degrading enzymes, β-1,4-endoglucanase and β-1,4-endoxylanase, is correlated with the abundance of their respective substrates in root exudates (Bell et al., 2019). The authors speculated that this transcriptional plasticity might be an evolutionary adaptation for host recognition and increased root invasion by this polyphagous species. Although, unlike *Pratylenchus coffeae*, PCN only reproduces on a few species belonging to the *Solanaceous* family, our transcriptome analysis identified not only β-1,4-endoglucanase genes (cellulases) but also expansin-like proteins as regulated in a host-dependent manner (Table 2), implying that root exudate tailored expression of cell wall-degrading genes may be a general adaptation for parasitism on plants rather than driven by host range. It would be interesting to measure whether tested root exudates differ in cellulose content, an organic compound shown to be an elicitor for β-1,4-endoglucanase (Bell et al., 2019). The genes with predicted function as parasitic effectors accounted for 40% (*G. pallida*) and 33% (*G. ellingtonae*) of all the functionally annotated DEGs in SSI hatching treatment, showing that not only cell wall-degrading enzymes, but also other types of parasitism genes, are regulated in a host-dependent manner. Lectins were the largest group of parasitism genes, which were significantly downregulated in SSI hatch in both *Globodera* spp. (Table 2). Recent studies showed that *G. rostochiensis* galectins and c-type lectins are present in the surface coat of *G. rostochiensis* juveniles, and their expression is localized and/or predicted to be in the hypodermis (Price, 2019). The authors speculate that hypodermically secreted lectin can bind to the nematode’s surface glycocalyx, which renders the nematode’s surface glycosylation invisible to the host. Alternatively, surface-released lectins could bind to the host damage-associated molecular pattern (DAMP) molecules, which most likely are abundantly produced during nematode migration through roots. Thus, lectins may suppress plant immunity by interfering with DAMP-triggered host defense response (Price, 2019). Two other SSI root exudate downregulated *Globodera* genes resembled the parasitic effectors: GROS_g07852, which is similar to *Meloidogyne javanica* transthyretin-like (MjTTL) gene linked to the attenuation of host resistance by interfering with reactive oxygen species (ROS) defense signaling (Lin et al., 2016); and GROS_g02373, which has a serine carboxypeptidase domain like *Radopholus similis* Rs-scp-1 effector, demonstrated to be important for the parasitic process (Huang et al., 2017). In addition, the expression of a gene similar to G20E03 (GPALN_015214) and members of a small effector family from *G. pallida* (GPALN_005097, GPALN_005105, GPALN_005081, and GPALN_005082) was reduced in nematodes hatched in *S. sisymbriifolium* root exudates, however, their function in nematode parasitism remains to be determined (Thorpe et al., 2014). Since the nematode’s capability to penetrate host roots and suppress plant immunity is essential for early parasitism, reduced expression of the above-mentioned genes may have played a role in the observed lower initial infection rate for SSI-hatched juveniles on a susceptible host. While SSI exudate strongly impacts the initial J2s ability to infect its host, the observed change in the parasitic behavior appear to be transient, and J2s successful in overcoming this barrier are capable of establishing a normal nematode life cycle in a susceptible host. Although mechanisms behind transcriptional control over parasitism genes remain unknown, the effect of SSI secretions may wear off once hatched juveniles are inoculated on the susceptible potato roots, and expression of DEGs is restored to similar levels as of those nematodes stimulated by potato exudates. In fact, the regulation of essential effector gene expression has been recently proposed to be the major determinant of the ability of four closely related *Globodera* spp. to infect potatoes (Sabeh et al., 2019).

The evidence suggests that selective breeding efforts for increased yield usually lead to trade-offs with defense mechanisms (Preece and Peñuelas, 2020). Likely, a crop domestication process has also adversely impacted beneficial traits associated with root exudation by reducing their natural diversity (Köllner et al., 2008; Degenhardt et al., 2009; Iannucci et al., 2017; Preece and Peñuelas, 2020). Perhaps, it is not surprising then that *S. sisymbriifolium*, as a wild potato relative, not only stimulates *Globodera* spp. hatch but also has a broader impact on nematode juveniles. We found several transcriptional changes in J2s exposed to SSI root exudate implying that these nematodes experience a stronger stress than J2s from potato root diffusates. For example, the expression of heat shock proteins and molecular chaperones (GROS_g09038 and GPALN_010189) that play a pivotal role in conferring stress tolerance (Pérez-Morales and Espinoza, 2015), was elevated in SSI-hatched juveniles. Furthermore, the genes involved in nematode antioxidant and detoxification pathways seem to be specifically enriched in our expression study. ROS are dangerous to nucleic acids, proteins, and lipids, and if uncontrolled, they can lead to cell death (Gillet et al., 2017). A gene coding for a protein similar to the THI4 thiazole biosynthetic enzyme (GPALN_010902), involved in the biosynthesis of B1 vitamin, was upregulated in SSI treatment. Since vitamin B1 is a strong antioxidant capable of counteracting damaging ROS accumulation, it was suggested to play an important ROS scavenging role for cyst nematodes (Duceppe et al., 2017). Our transcriptomic analysis also showed elevated expression levels for glutathione synthetases (GROS_g09038 and GROS_g09037), key enzymes in the biosynthesis of glutathione, which play a critical role in protecting cells from oxidative damage and the toxicity of xenobiotics (Forman et al., 2009). Xenobiotic detoxification in *Caenorhabditis elegans* is usually carried out in three phases. Phase I is a biotransformation of the original compound to introduce or expose functional groups through either oxidation, reduction, or hydrolysis. Phase II is a conjugation of the substrate with a large, water-soluble group, to facilitate excretion. Enzymes such as UDP-glucuronosyltransferases (UGTs), sulfotransferases (SULTs), glutathione S-transferases (GSTs), and N-acetyltransferases (NATs) facilitate this process. Phase III exports xenobiotics and metabolites out of the cell *via* ABC-binding cassette (ABC) transporters (Hartman et al., 2021). Our RNA-seq data for SSI-hatched J2s show differential expression of genes involved in all three phases including oxidoreductase (GPALN_001038), two UDP-glucuronosyltransferases (GPALN_002696 and GPALN_003745), and an ABC transporter (GPALN_005765). Furthermore, one of the SSI-upregulated genes, GPALN_002494, has been recently shown to be induced in *G. pallida* after exposure to a nematicide Dazomet (Lennon, 2018). These results indicate that the SSI exudates may contain toxic metabolites that can have the potential to be used as biopesticides. The production of phytoalexins, a class of toxic low-molecular-mass secondary metabolites, in response to nematode infection, has been reported previously (Chitwood, 2002; Ahuja et al., 2012). Given that the root exudates obtained for this study were collected from plants not challenged with nematodes, the potential toxicity to the J2s might have been contributed by a different class of compounds. Many plant species contain constitutively produced phytochemicals that are involved in the direct defense response system called phytoanticipins. *Solanaceous* spp. contain a diverse group of phytoanticipins called glycoalkaloids. Interestingly, a recent study on four steroidal glycosides (α-solamargine, α-solamarine, α-solasonine, and solasodine) present in *Solanaceous* species showed their deleterious impact on *G. pallida* hatch, development, and reproduction (Pillai and Dandurand, 2021a). Although the presence of steroidal glycosides in SSI exudates including those mentioned above remains to be determined, these types of complex secondary metabolites may at least in part influence the expression of J2 detoxification genes once animals are hatched in SSI root exudate.

The current body of knowledge does not support the hypothesis that host resistance influences *Globodera* spp. chemotaxis (Torto et al., 2018). However, our data showed that hatching conditions may have an impact on general nematode motility (Figure 1). Phospholipase A2 (PLA2) catalyzes the hydrolysis of the sn-2 position of membrane glycerophospholipids to liberate arachidonic acid (AA), which is a chemical messenger released by muscles (Balsinde et al., 2002). In *C. elegans*, AA is involved in the neurotransmission necessary for locomotion (Lesa et al., 2003; Schmeisser et al., 2019), therefore, the reduced expression of PLA2-like genes (GPALN_006585 and GPALN_006587) in SSI hatching may potentially explain an adverse impact on nematode motility.

It is worth noticing that the impact of SSI root exudates is much more evident on *G. ellingtonae* than *G. pallida* across all our analyses. In line with a greater reduction of infection rate, significant impact on reproduction and number of eggs per cyst as well as greater altered motility, the RNA-seq analysis identified not only a larger number of DEGs (Figure 6) but also a larger magnitude of expression level change (Supplementary Data Sheets 1, 2). It has been observed that the *Globodera* spp. respond differentially to the hatching process. *Globodera pallida* has been shown to be less responsive to chemicals inducing hatch compared with *G. rostochiensis* (Byrne et al., 2001), thus the same may hold true for the effect of other, yet uncharacterized, compounds of SSI root exudate. SSI root exudate specific upregulation of *G. ellingtonae* gene containing a seven-transmembrane G-protein-coupled receptor domain (7TM GPCRs; GROS_g07382) typical for the Sra superfamily of chemoreceptors in *C. elegans* (Thomas and Robertson, 2008), which was not observed for the *G. pallida* ortholog, could support this hypothesis.

It is possible that the criteria we set to identify DEGs were too stringent and genes with lower changes in expression, which were not discussed here [(log_2_FC) < 1.5; Supplementary Tables 3, 4], may still have a biological significance. Although a comparative gene expression from J2s exposed to susceptible and resistant potato root exudates yielded only one gene with altered expression for each *Globodera* spp., the alignment of DS *vs*. SSI and INN *vs*. SSI comparisons showed many DEGs that were unique for each group evaluation (Figures 6C,D). This implies that a larger number of nematode genes is differentially regulated in response to plant exudates, and even though the magnitude of expression change is lower, these changes collectively may alter nematode behavior.

In conclusion, the significance of root exudates as a buffer zone that protects plants against infection may be underestimated. In this study, we demonstrated that nematode behavior and its ability to infect plants can be differentially regulated in a host-dependent manner even prior to root invasion. Little is known how plant-delivered metabolites impact nematodes at the molecular level. Our transcriptomic data pointed to at least two possible root exudate-mediated mechanisms that support our biological observations. First, the host type influences the expression level of some nematode parasitism genes. If parasitic gene activators from root exudates are identified in the future, breeding efforts to obtain potato varieties with low expression of such elicitors may lead to enhanced nematode resistance (Sikder and Vestergård, 2020). Second, SSI root exudate might contain phytoanticipins with a nematicidal potential. It is not uncommon that chemically synthesized nematicides show adverse environmental and toxic effects, and therefore, many are no longer available or banned (Khalil, 2013). An increased understanding of host-specific nematode transcriptional responses and phytotoxic activity from secretions of wild potato relatives will benefit the development of environmentally sustainable control methods. The modulation of the chemical interactions between plant roots and plant-parasitic nematodes has the potential to be a novel management strategy for plant parasitic nematodes.

## Data Availability Statement

The datasets presented in this study can be found in online repositories. The names of the repository/repositories and accession number(s) can be found below: https://www.ncbi.nlm.nih.gov/, PRJNA788476.

## Author Contributions

JK, SP, and L-MD were involved in the conceptualization and experimental design. JK, SP, and GR are responsible for conducting experiments and analyzing the data. JK wrote the draft of the original manuscript. L-MD, JCK, AC, and FX obtained funding for this research. All authors reviewed the manuscript and have approved it for publication.

## Conflict of Interest

The authors declare that the research was conducted in the absence of any commercial or financial relationships that could be construed as a potential conflict of interest.

## Publisher’s Note

All claims expressed in this article are solely those of the authors and do not necessarily represent those of their affiliated organizations, or those of the publisher, the editors and the reviewers. Any product that may be evaluated in this article, or claim that may be made by its manufacturer, is not guaranteed or endorsed by the publisher.
