# Supplementary material for: Belowground Chemical Interactions: An Insight Into Host-Specific Behavior of Globodera spp. Hatched in Root Exudates From Potato and Its Wild Relative, Solanum sisymbriifolium
Source: Front Plant Sci. 2022 Jan 12;12:802622. doi: 10.3389/fpls.2021.802622 (PMC8791010; doi:10.3389/fpls.2021.802622)
Supplement: Supplementary file 1 [file Table_1.PDF]

Supplementary Table 1. Comparison of percent egg viability for *G. pallida* and *G. ellingtonae* depending on the original hatching treatment (DS – Desirée, INN - Innovator, SSI – *S. sisymbriifolium*) was determined using Meldola's Blue method. Each value represents the mean ± standard error of 8 replicates. Means followed by the same letter designation are not significantly different as determined using ANOVA followed by Tukey's HSD test at $p<0.05$.

treatment	*G. pallida*	*G. ellingtonae*
**DS**	22 ± 2[a]	31 ± 4[b]
**INN**	18 ± 3[a]	32 ± 5[b]
**SSI**	19 ± 5[a]	32 ± 5[b]
