# Supplementary material for: Belowground Chemical Interactions: An Insight Into Host-Specific Behavior of Globodera spp. Hatched in Root Exudates From Potato and Its Wild Relative, Solanum sisymbriifolium
Source: Front Plant Sci. 2022 Jan 12;12:802622. doi: 10.3389/fpls.2021.802622 (PMC8791010; doi:10.3389/fpls.2021.802622)
Supplement: Supplementary file 2 [file Table_2.PDF]

Supplementary Table 2 Primers used for qRT-PCR

**Name**	**Sequence (5' → 3')**	**Purpose**	**Reference**
EF1αF	AACATCTCTGTGAAGGACATTCG	qRT-PCR, internal reference gene	Thorpe, 2012
EF1αR	TCTCCTTAAGTTCGGCGAATTTGC	qRT-PCR, internal reference gene	Thorpe, 2012
G_014919 RT_F	GGTCAAGGAATTGGAGGAGAAA	qRT-PCR, GPALN_014919 gene	*this study*
G_014919 RT_R	TGGCACACTGCTGAAGAAA	qRT-PCR, GPALN_014919 gene	*this study*
G_006587 RT_F	GTGGGAGTGACCGCTTTAATA	qRT-PCR, GPALN_006587 gene	*this study*
G_006587 RT_R	GGCTTGTGCCGAATAACATAG	qRT-PCR, GPALN_006587 gene	*this study*
G_003867 RT_F	ACAGTCCTTAGCAAACGTAACA	qRT-PCR, GPALN_003867 gene	*this study*
G_003867 RT_R	AGAAATAGTCCAGCCGAAACC	qRT-PCR, GPALN_003867 gene	*this study*
